# Hemophagocytic Lymphohistiocytosis Secondary to Dengue Fever in a Pediatric Patient: A Case Report

**DOI:** 10.7759/cureus.59165

**Published:** 2024-04-27

**Authors:** Lakshiya Ramamoorthy, Nithila Sivakumar, Lakshmi Murugesan, Arul Kumar

**Affiliations:** 1 Internal Medicine, Madras Medical College, Chennai, IND; 2 Pediatric Medicine, Government Tiruppur Medical College and Hospital, Tiruppur, IND

**Keywords:** cytopenia, hemophagocytes, dengue hemorrhagic fever, pancytopenia, hemophagocytic lymphohistiocytosis, dengue fever

## Abstract

Dengue fever, an arboviral illness, exhibits a broad range of symptoms, ranging from flu-like symptoms to serious hemorrhagic complications. Hemophagocytic lymphohistiocytosis (HLH) is an uncommon pathological state caused by excessive activation of the immune system, culminating in organ dysfunction. HLH can be primary or secondary, with infection being the most common cause. The association between dengue fever and dengue-induced HLH is becoming widely acknowledged as a lethal complication.

We present the case of a two-year-old male child referred for the management of dengue infection. The patient’s condition failed to ameliorate despite appropriate treatment. On further investigation, he was diagnosed with HLH. Following the initiation of steroid therapy, the patient demonstrated gradual improvement with normalization of laboratory parameters.

Differentiating between HLH and severe dengue hemorrhagic fever poses a significant challenge, emphasizing the importance of prompt diagnosis for favorable outcomes. Early identification and commencement of corticosteroid therapy are imperative for successful management.

## Introduction

Dengue fever, a prevalent disease in tropical regions, stems from an infection by a positive-sense, single-stranded RNA virus. This virus is part of the Flaviviridae family. There are four distinct serotypes (DEN1, DEN2, DEN3, DEN4), all transmitted by the *Aedes* mosquito. Simple dengue fever usually includes symptoms such as headache, high fever, rash, muscle ache, and joint pain, with a mortality rate below 1%. On the other hand, severe manifestations of dengue, such as dengue hemorrhagic fever (DHF) and dengue shock syndrome, are distinguished by thrombocytopenia, increased vascular permeability, and hypotension [1,2].

Hemophagocytic lymphohistiocytosis (HLH) is a life-threatening autoimmune disorder marked by uninhibited stimulation and proliferation of cytotoxic T lymphocytes and histiocytes, leading to excessive release of immune mediators, and causing damage to cells and organs. HLH can be either familial (primary) or acquired (secondary). Primary HLH is more common than secondary HLH, with an incidence rate of 1.2 per million children per year [3]. However, there are currently no comprehensive reports thoroughly characterizing the incidence associated with each etiology of secondary HLH. One retrospective study conducted in the United States, which pooled data from 2006 to 2019 to infer trends, found that infections accounted for 24.3% of secondary HLH cases. Among these infections, viral infections (21.6%) were the most common [1]. Nevertheless, studies focusing on determining the burden of HLH specifically in the context of DHF are yet to be conducted.

The inheritance pattern is typically autosomal recessive for familial forms of HLH, with 40-60% of mutations occurring in the *PRF1* and *UNC13D* genes, along with the involvement of the *STX11* and *STXBP2* genes [4]. Secondary HLH is typically caused by viral infections, immunodeficiency states, autoimmune diseases, or malignancy. While the Epstein-Barr virus is the predominant viral trigger for HLH, dengue-induced HLH has been increasingly reported, suggesting a potentially serious manifestation of the illness [5]. The mortality rates for secondary HLH fluctuate between 30% and 40% during the first two months following diagnosis [6]. Here, we present a case of dengue-induced HLH which resolved following prompt diagnosis and adequate treatment.

## Case presentation

A two-year-old male child was brought to the emergency department at Government Medical College and Hospital, Tiruppur, on February 23, 2022, under the referral of a private general practitioner for further management of dengue infection. The diagnosis was confirmed using a rapid NS1 antigen card test, prompted by the child’s persistent high fever and refusal of feeds over the preceding five days, as well as a noticeable decrease in activity levels reported by his mother. Additionally, no other notable symptoms were evident upon presentation, and the child’s past medical history was unremarkable. He had received all appropriate vaccinations for his age and had achieved developmental milestones consistent with his age group.

Upon physical examination, the child appeared toxic and visibly unwell, with a recorded temperature of 102.4°F. His vital signs include a blood pressure of 86/62 mmHg, a heart rate of 141 beats per minute, a respiratory rate of 37 breaths per minute, and an oxygen saturation that remained stable at 97% in ambient air. A thorough cardiopulmonary examination yielded normal findings, whereas an abdominal examination revealed a firm, non-tender liver palpable 5 cm beneath the right costal margin with non-tender splenomegaly (4 cm beneath the left costal margin). The remainder of the physical examination revealed no significant findings.

Laboratory investigations unveiled leukopenia (leukocyte count: 3.7 × 10^9^/L), anemia (hemoglobin: 5.7 g/dL), and thrombocytopenia (platelet count: 18 × 10^3^/µL). Dengue NS1 testing returned positive results, while IgM scrub typhus testing yielded negative results. Additionally, no malarial parasites were identified on a Giemsa-stained smear examination. The tests were performed to rule out possible differential diagnoses.

Initial management included intravenous fluid therapy and antipyretic medication. However, despite these interventions, the child’s clinical condition continued to deteriorate, characterized by persistent high-grade fever and the development of spontaneous bruising at various sites, including the forehead, elbow, and behind the ears. Further investigations (Table 1) on February 24, 2022, revealed a significant decline in all three cell lines, with packed red blood cells, whole blood, and platelet transfusions proving ineffective in improving cell counts. Other investigations which were done are presented in Table 2.

**Table 1:** Comparison of serial investigations.

Parameters	Reference range	Values				
		February 23, 2022	February 24, 2022	February 25, 2022	February 26, 2022	February 27, 2022
Total count	6–17 × 10^9^/L	3,600	1,700	1,800	1,800	1,900
Hemoglobin	10.9–15.0 mg/dL	5.7	4.6	4.6	4.2	6.7
Platelet count	250–600 × 10^3^/µL	18,000	15,000	67,000	10,000	35,000

**Table 2:** Other relevant investigations. CRP = C-reactive protein; AST = aspartate aminotransferase; ALT = alanine aminotransferase

Variable	Reference range	Upon presentation
CRP	1.8–4.1 mg/dL	21.2
Sodium	136–145 mEq/L	132
Potassium	3.5–5 mEq/L	3.7
Total serum bilirubin	0–1.5 mg/dL	0.8
AST	15–46 U/L	16
ALT	8–36 U/L	19
Serum albumin	3.5–5.5 g/dL	2.1
Serum triglyceride	48–126 mg/dL	352
Corrected reticulocyte count	0.5–2.5%	0.5%

**Figure 1:**
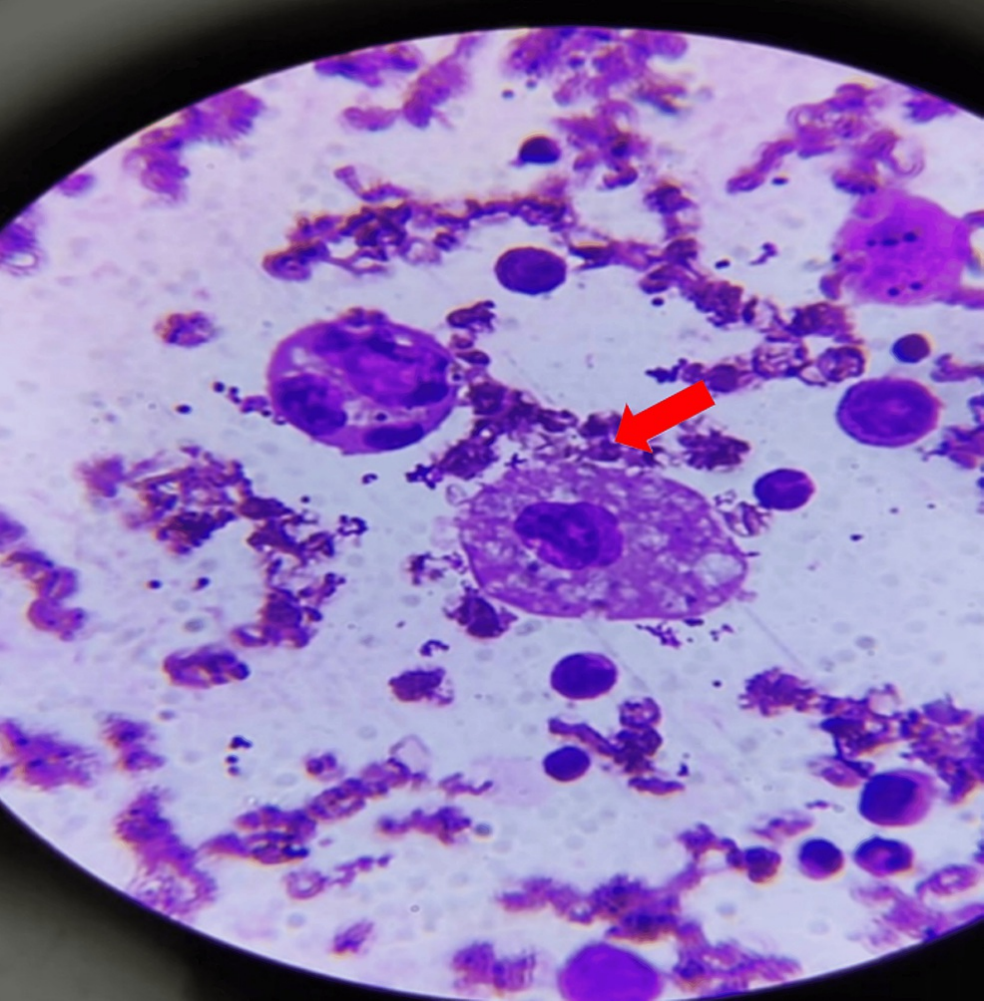
Bone marrow aspiration and biopsy. Bone marrow aspirate revealing macrophage with significant hemophagocytic activity (marked with an arrow).

**Figure 2:**
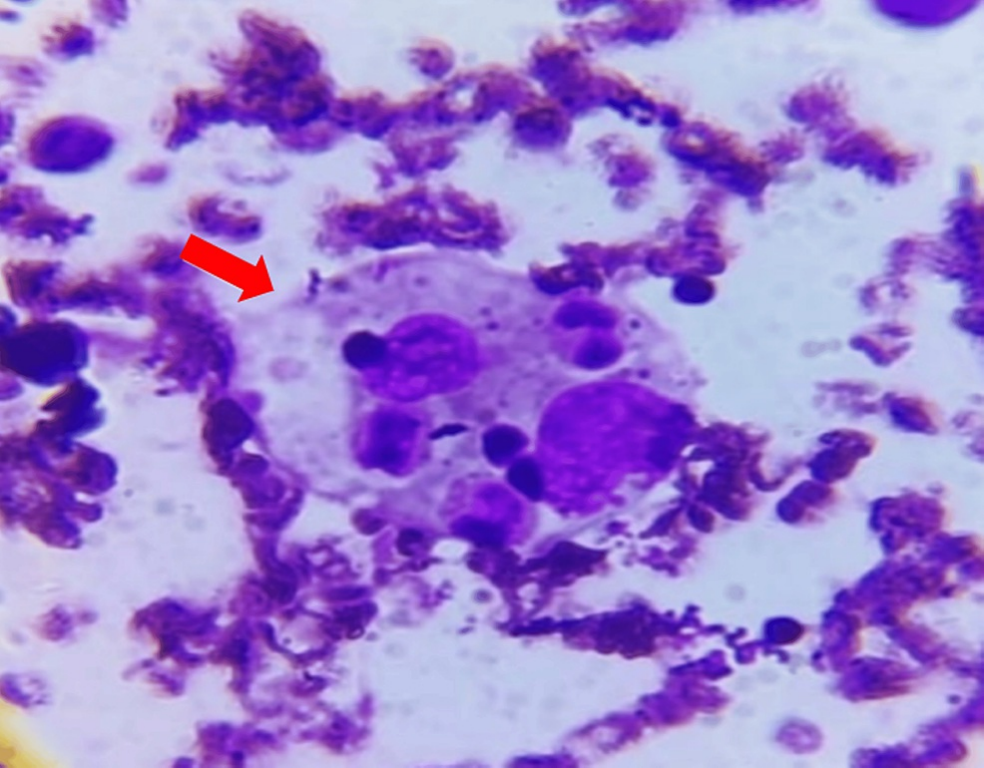
High-power field of the bone marrow aspirate. The image reveals evident histiocyte activity with the engulfment of a neutrophil and mature erythrocytes, indicating the presence of hemophagocytic lymphohistiocytosis (marked with an arrow).

Subsequent peripheral smear analysis indicated microcytic hypochromic anemia, leukopenia, and thrombocytopenia with the presence of giant platelets. Abdominal ultrasound imaging revealed hepatomegaly (10.6 cm) with normal echotexture and splenomegaly (10 cm), while triglyceride levels were 352 mg/dL. Given the clinical and laboratory criteria for HLH, a bone marrow biopsy was performed, confirming trilineage hematopoiesis with increased histiocytes and the presence of hemophagocytes (Figures 1, 2).

Treatment was initiated in accordance with established Indian Academy of Pediatrics guidelines, which included the administration of intravenous dexamethasone (10 mg/m^2^) in divided doses, empirical antibiotic therapy, and blood component transfusions to address the declining cell counts. Within 24 hours of initiating treatment, the child’s fever subsided, marking an initial positive response to therapy. As the child’s condition stabilized, intravenous steroid therapy was transitioned to oral dexamethasone, with a gradual tapering regimen over 21 days. Subsequently, the patient demonstrated significant improvement and an excellent recovery. After being discharged, the child was reassessed and found to have normal blood counts, indicating a return to normal health.

## Discussion

Dengue fever is a rare but notable trigger for HLH, particularly in individuals exhibiting unexplained systemic inflammatory response syndrome, characterized by prolonged fever lasting over eight days, cytopenia, malaise, and hepatosplenomegaly [7]. Although sepsis is a primary consideration in children exhibiting persistent fever after recovering from dengue fever, clinicians should maintain a high suspicion for HLH in these cases and initiate early evaluation [8]. HLH is a severe and uncommon condition, predominantly affecting infants up to the age of 18 months. However, it can also occur in children and adults. The initial presentation of HLH often resembles that of more prevalent illnesses, making diagnosis challenging. The disease manifests with fever and features suggesting the involvement of multiple organ systems [9].

Viral infections are frequently implicated in HLH pathogenesis, although the precise mechanisms remain unclear. Viruses exert potent effects on the immune system, disrupting cytokine balance, immune recognition, and apoptotic pathways. Some theories propose that aberrant proliferation and activation of T cells may trigger macrophage activation and impaired phagocyte destruction. Others suggest the involvement of perforin and natural killer (NK) cells, with perforin deficiency weakening cellular defenses and reducing NK cell activity, thereby enhancing T-cell function and cytokine production. These processes, often observed in viral diseases, result in prolonged macrophage activation. Although macrophages ingest infected leukocytes, they are ineffective at eliminating them. Active T cells, along with numerous activated macrophages, escalate inflammatory cytokine production, exacerbating bone marrow and tissue damage.

During acute dengue infection, invariant NK T cells become activated, producing interferon-gamma and granulocyte-macrophage colony-stimulating factor upon stimulation. CD25 is a useful inflammatory marker in the workup for HLH [3]. Comprehensive research is necessary to elucidate the intricate pathogenesis of HLH and its association with dengue fever [10].

The confirmation of HLH diagnosis typically requires the presence of at least five out of the eight specified features, namely, fever ≥38.5°C; splenomegaly; peripheral blood cytopenia; hypertriglyceridemia and/or hypofibrinogenemia; hemophagocytosis in bone marrow, spleen, lymph node, or liver; low or absent NK cell activity; ferritin >500 ng/mL; and elevated soluble CD25 (soluble IL-2 receptor alpha) with two standard deviations above age-adjusted, laboratory-specific norms [11]. Our patient demonstrated fever ≥38.5°C, splenomegaly, peripheral blood cytopenia, hypertriglyceridemia, and hemophagocytosis, thereby confirming the diagnosis.

The treatment strategy proposed during the second global assembly of the Histiocyte Society in 2004 advocates for an initial therapeutic regimen spanning eight weeks, involving corticosteroids, etoposide, and cyclosporine A. Simultaneous administration of anti-infective therapy (intrathecal methotrexate in specific patients) aims to eliminate the instigating factor and address the compromised immune system. For individuals with familial disease, a confirmed molecular diagnosis, or severe, persistent, or reactivated disease, hematopoietic stem cell transplantation is recommended. In instances of less severe HLH manifestations, isolated steroid therapy or concomitant immunoglobulin administration may be adequate to combat immune system hyperactivation. Nevertheless, even seemingly mild cases can swiftly worsen [12].

Secondary HLH represents a swiftly fatal condition. Without intervention, dengue-associated HLH carries a high mortality rate. Most fatalities result from bacterial or fungal infections, mainly because of persistent neutropenia or widespread organ dysfunction. While some cases of dengue-associated HLH miraculously recuperate with conservative therapy alone, pulse doses of methylprednisolone or dexamethasone are commonly employed to quell the hyperinflammatory condition [13].

In summary, early clinical recognition and timely diagnosis are crucial for the appropriate management of HLH [14]. Intravenous dexamethasone was promptly commenced upon heightened clinical suspicion. Upon achieving hemodynamic stability, intravenous steroids were transitioned to oral administration and gradually tapered over 21 days. The patient showed an excellent treatment response to steroid therapy.

## Conclusions

The above study highlights the significance of identifying HLH as a possible sequela in cases where prolonged febrile illness and cytopenias develop following recovery from dengue infection, particularly in individuals presenting with distinctive signs such as hepatosplenomegaly. Early detection and timely administration of suitable immunosuppressive pharmacotherapy, such as intravenous corticosteroids, can lead to a positive outcome and favorable prognosis. Despite the exploration of various immunosuppressive medications and hematopoietic stem cell transplantation modalities, our case report and emerging evidence advocate for the utilization of dexamethasone to mitigate the virus-induced inflammatory cascade, thereby serving as an efficacious therapeutic approach for HLH secondary to dengue infection.
